# *mer-*Triaqua­(1,10-phenanthroline-κ^2^ *N*,*N*′)(sulfato-κ*O*)magnesium(II)

**DOI:** 10.1107/S1600536808009938

**Published:** 2008-04-18

**Authors:** Ling Zhu, Jing Huang, Si-Ying Han, Zhe An

**Affiliations:** a School of Pharmaceutical Science, Harbin Medical University, Harbin 150086, People’s Republic of China

## Abstract

In the title compound, [Mg(SO_4_)(C_12_H_8_N_2_)(H_2_O)_3_], the Mg^II^ centre exhibits a slightly distorted octa­hedral coordination environment defined by two N atoms from a 1,10-phenanthroline mol­ecule, one O atom from a sulfate dianion and three meridionally arranged O atoms from coordinated water mol­ecules. The crystal structure involves intra- and intermolecular O—H⋯O hydrogen bonds.

## Related literature

For copper(II), zinc(II) and cadmium(II) complexes of phenanthroline, see: Xu *et al.* (2003 ▶); Zhang *et al.* (1999 ▶).
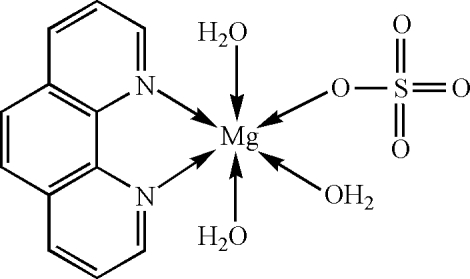


## Experimental

### 

#### Crystal data

[Mg(SO_4_)(C_12_H_8_N_2_)(H_2_O)_3_]
*M* *_r_* = 354.62
Monoclinic, 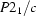

*a* = 11.968 (2) Å
*b* = 10.025 (2) Å
*c* = 13.798 (3) Å
β = 113.53 (3)°
*V* = 1517.8 (6) Å^3^
*Z* = 4
Mo *K*α radiation
μ = 0.29 mm^−1^
*T* = 295 (2) K
0.36 × 0.28 × 0.20 mm

#### Data collection

Rigaku R-AXIS RAPID diffractometer
Absorption correction: multi-scan (*ABSCOR*; Higashi, 1995 ▶) *T*_min_ = 0.902, *T*_max_ = 0.944
14532 measured reflections
3454 independent reflections
2941 reflections with *I* > 2σ(*I*)
*R*_int_ = 0.023

#### Refinement

*R*[*F* ^2^ > 2σ(*F* ^2^)] = 0.033
*wR*(*F* ^2^) = 0.098
*S* = 1.07
3454 reflections
226 parameters
9 restraints
H atoms treated by a mixture of independent and constrained refinement
Δρ_max_ = 0.39 e Å^−3^
Δρ_min_ = −0.30 e Å^−3^

### 

Data collection: *RAPID-AUTO* (Rigaku, 1998 ▶); cell refinement: *RAPID-AUTO*; data reduction: *CrystalStructure* (Rigaku/MSC, 2002 ▶); program(s) used to solve structure: *SHELXS97* (Sheldrick, 2008 ▶); program(s) used to refine structure: *SHELXL97* (Sheldrick, 2008 ▶); molecular graphics: *SHELXTL* (Sheldrick, 2008 ▶); software used to prepare material for publication: *SHELXL97*.

## Supplementary Material

Crystal structure: contains datablocks I, global. DOI: 10.1107/S1600536808009938/im2060sup1.cif

Structure factors: contains datablocks I. DOI: 10.1107/S1600536808009938/im2060Isup2.hkl

Additional supplementary materials:  crystallographic information; 3D view; checkCIF report

## Figures and Tables

**Table 1:** Hydrogen-bond geometry (Å, °)

*D*—H⋯*A*	*D*—H	H⋯*A*	*D*⋯*A*	*D*—H⋯*A*
O2*W*—H2*W*1⋯O3^i^	0.850 (9)	1.891 (11)	2.7170 (17)	163.8 (16)
O3*W*—H3*W*1⋯O2^ii^	0.848 (9)	1.906 (10)	2.7375 (17)	166.2 (17)
O1*W*—H1*W*1⋯O1^ii^	0.864 (9)	1.862 (10)	2.7235 (17)	174.8 (17)
O2*W*—H2*W*2⋯O2^iii^	0.852 (9)	1.873 (10)	2.7232 (17)	175.4 (19)
O1*W*—H1*W*2⋯O2	0.859 (19)	1.862 (19)	2.7030 (17)	166.0 (19)
O3*W*—H3*W*2⋯O3^iii^	0.843 (9)	1.965 (10)	2.7961 (19)	168.5 (17)

Symmetry codes: (i) 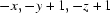; (ii) 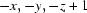; (iii) 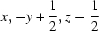.

**Fig. 1.**
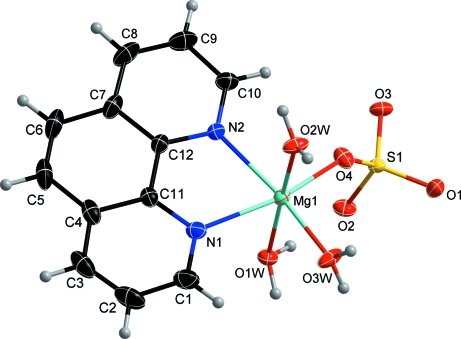
Molecular structure of (I), showing 30% displacement ellipsoids level.

**Fig. 2.**
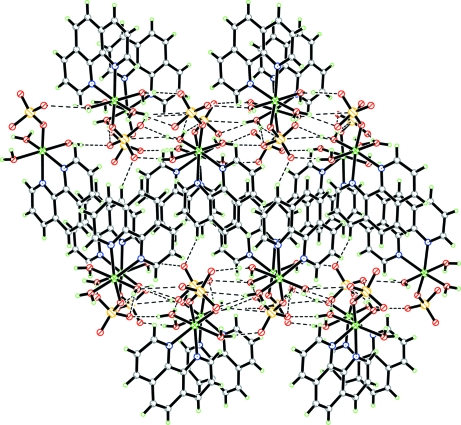
Packing diagram of (I).

## supplementary crystallographic information

### Comment

1,10-Phenanthroline (phen) is one of the most commonly used aromatic N,N' chelating ligands and has in form of several functionalized derivatives been widely used in the construction of supramolecular architectures.

Copper(II), zinc(II) and cadmium(II) derivatives of phen have been reported (Xu *et al.*, 2003; Zhang *et al.* 1999).

As a continuation of these studies, we now report the crystal structure of the title complex, (I).

As illustrated in Fig. 1, the Mg(II) ion is surrounded by two N atoms from the phen ligand and four O atoms from three meridionally arranged H_2_O molecules and one sulfato group to form distorted MgN_2_O_4_ octahedron. The Mg—O and Mg—N bond lengths are in the noramal range of 2.033 (1)–2.086 (1) and 2.210 (1)–2.234 (2) Å, respectively. The units are connected by O—H···O hydrogen bonds to produce a complex three dimensional supramolecular network, shown in figure 2.

### Experimental

1,10-phenanthroline (0.05 g, 0.25 mmol) was dissolved in a water-DMF mixture (1:1 *v*/*v*, 50 ml), and MgSO_4_ × 7 H_2_O (0.06 g, 0.25 mmol) was added to the solution. The resulting mixture was stirred at room temperature for 12 h and filtered. Colorless single crystals of (I) were obtained from the solution after several weeks.

### Refinement

The carbon-bound H atoms were positioned geometrically (C—H = 0.93–0.97 Å) and refined as riding with *U*_iso_(H) = 1.2*U*_eq_(C). The O-bound H atoms were located in a difference map and refined with a distance restraint of 0.85 (1) Å and the constraint *U*_iso_(H) = 1.5*U*_eq_(O).

### Crystal data

**Table:**

[Mg(SO_4_)(C_12_H_8_N_2_)(H_2_O)_3_]	*F*_000_ = 736
*M**_r_* = 354.62	*D*_x_ = 1.552 Mg m^−^^3^
Monoclinic, *P*2_1_/*c*	Mo *K*α radiation λ = 0.71073 Å
Hall symbol: -P 2ybc	Cell parameters from 11791 reflections
*a* = 11.968 (2) Å	θ = 3.0–27.5º
*b* = 10.025 (2) Å	µ = 0.29 mm^−^^1^
*c* = 13.798 (3) Å	*T* = 295 (2) K
β = 113.53 (3)º	Prism, colorless
*V* = 1517.8 (6) Å^3^	0.36 × 0.28 × 0.20 mm
*Z* = 4	

### Data collection

**Table:**

Rigaku R-AXIS RAPID diffractometer	3454 independent reflections
Radiation source: fine-focus sealed tube	2941 reflections with *I* > 2σ(*I*)
Monochromator: graphite	*R*_int_ = 0.023
Detector resolution: 10.000 pixels mm^-1^	θ_max_ = 27.5º
*T* = 295(2) K	θ_min_ = 3.0º
ω scans	*h* = −15→15
Absorption correction: multi-scan(ABSCOR; Higashi, 1995)	*k* = −12→13
*T*_min_ = 0.902, *T*_max_ = 0.944	*l* = −17→17
14532 measured reflections	

### Refinement

**Table:**

Refinement on *F*^2^	Secondary atom site location: difference Fourier map
Least-squares matrix: full	Hydrogen site location: inferred from neighbouring sites
*R*[*F*^2^ > 2σ(*F*^2^)] = 0.033	H atoms treated by a mixture of independent and constrained refinement
*wR*(*F*^2^) = 0.098	*w* = 1/[σ^2^(*F*_o_^2^) + (0.059*P*)^2^ + 0.2998*P*] where *P* = (*F*_o_^2^ + 2*F*_c_^2^)/3
*S* = 1.07	(Δ/σ)_max_ = 0.001
3454 reflections	Δρ_max_ = 0.39 e Å^−^^3^
226 parameters	Δρ_min_ = −0.30 e Å^−^^3^
9 restraints	Extinction correction: none
Primary atom site location: structure-invariant direct methods	

### Special details

**Table:**

Geometry. All e.s.d.'s (except the e.s.d. in the dihedral angle between two l.s. planes) are estimated using the full covariance matrix. The cell e.s.d.'s are taken into account individually in the estimation of e.s.d.'s in distances, angles and torsion angles; correlations between e.s.d.'s in cell parameters are only used when they are defined by crystal symmetry. An approximate (isotropic) treatment of cell e.s.d.'s is used for estimating e.s.d.'s involving l.s. planes.
Refinement. Refinement of *F*^2^ against ALL reflections. The weighted *R*-factor *wR* and goodness of fit *S* are based on *F*^2^, conventional *R*-factors *R* are based on *F*, with *F* set to zero for negative *F*^2^. The threshold expression of *F*^2^ > σ(*F*^2^) is used only for calculating *R*-factors(gt) *etc*. and is not relevant to the choice of reflections for refinement. *R*-factors based on *F*^2^ are statistically about twice as large as those based on *F*, and *R*- factors based on ALL data will be even larger.

### Fractional atomic coordinates and isotropic or equivalent isotropic displacement parameters (Å^2^)

**Table:**

	*x*	*y*	*z*	*U*_iso_*/*U*_eq_	
Mg1	0.13726 (4)	0.25963 (5)	0.47420 (4)	0.02612 (13)	
S1	−0.06777 (3)	0.22093 (3)	0.57725 (3)	0.02562 (11)	
O1	−0.17699 (10)	0.15328 (13)	0.50549 (11)	0.0455 (3)	
O1W	0.19239 (10)	0.09656 (11)	0.57638 (9)	0.0334 (2)	
O2	0.01644 (11)	0.12166 (11)	0.65134 (9)	0.0366 (3)	
O2W	0.08603 (12)	0.40809 (11)	0.36365 (9)	0.0391 (3)	
O3	−0.09964 (11)	0.32153 (11)	0.63949 (9)	0.0384 (3)	
O3W	0.06431 (12)	0.13366 (12)	0.34794 (9)	0.0446 (3)	
O4	−0.00451 (10)	0.28537 (12)	0.51813 (9)	0.0382 (3)	
N1	0.32491 (12)	0.24769 (13)	0.47706 (11)	0.0356 (3)	
N2	0.24343 (11)	0.41322 (12)	0.58883 (10)	0.0298 (3)	
C1	0.36509 (19)	0.1648 (2)	0.42311 (17)	0.0524 (5)	
H1	0.3098	0.1075	0.3744	0.063*	
C2	0.4875 (2)	0.1602 (2)	0.4366 (2)	0.0659 (6)	
H2	0.5124	0.0993	0.3983	0.079*	
C3	0.56924 (19)	0.2443 (2)	0.5052 (2)	0.0598 (6)	
H3	0.6504	0.2424	0.5138	0.072*	
C4	0.53087 (15)	0.3344 (2)	0.56323 (15)	0.0440 (4)	
C5	0.61079 (16)	0.4266 (2)	0.63750 (18)	0.0583 (6)	
H5	0.6930	0.4274	0.6495	0.070*	
C6	0.56954 (18)	0.5119 (2)	0.69028 (16)	0.0587 (6)	
H6	0.6237	0.5711	0.7381	0.070*	
C7	0.44359 (16)	0.51409 (19)	0.67458 (13)	0.0443 (4)	
C8	0.3943 (2)	0.6039 (2)	0.72430 (16)	0.0599 (6)	
H8	0.4441	0.6679	0.7702	0.072*	
C9	0.2739 (2)	0.5983 (2)	0.70592 (16)	0.0574 (5)	
H9	0.2405	0.6589	0.7379	0.069*	
C10	0.20141 (16)	0.50001 (17)	0.63810 (13)	0.0407 (4)	
H10	0.1196	0.4955	0.6271	0.049*	
C11	0.40669 (13)	0.33127 (16)	0.54670 (12)	0.0324 (3)	
C12	0.36302 (13)	0.42130 (15)	0.60482 (11)	0.0311 (3)	
H2W1	0.0974 (17)	0.4912 (10)	0.3757 (13)	0.047*	
H3W1	0.0497 (17)	0.0531 (11)	0.3577 (14)	0.047*	
H1W1	0.1920 (16)	0.0180 (12)	0.5506 (14)	0.047*	
H2W2	0.0668 (18)	0.3949 (17)	0.2979 (8)	0.047*	
H1W2	0.1422 (15)	0.0933 (18)	0.6067 (14)	0.047*	
H3W2	0.0186 (15)	0.1583 (17)	0.2863 (9)	0.047*	

### Atomic displacement parameters (Å^2^)

**Table:**

	*U*^11^	*U*^22^	*U*^33^	*U*^12^	*U*^13^	*U*^23^
Mg1	0.0285 (3)	0.0215 (2)	0.0276 (3)	−0.00238 (18)	0.0104 (2)	−0.00030 (17)
S1	0.03023 (19)	0.01845 (18)	0.03043 (19)	−0.00086 (12)	0.01448 (15)	−0.00115 (12)
O1	0.0335 (6)	0.0388 (7)	0.0627 (8)	−0.0073 (5)	0.0177 (6)	−0.0200 (6)
O1W	0.0371 (6)	0.0265 (5)	0.0364 (6)	0.0044 (4)	0.0145 (5)	0.0030 (4)
O2	0.0492 (6)	0.0277 (6)	0.0367 (6)	0.0085 (5)	0.0212 (5)	0.0088 (4)
O2W	0.0656 (8)	0.0205 (5)	0.0299 (5)	−0.0014 (5)	0.0177 (5)	0.0015 (4)
O3	0.0519 (7)	0.0244 (5)	0.0410 (6)	0.0034 (5)	0.0206 (5)	−0.0070 (4)
O3W	0.0656 (8)	0.0230 (6)	0.0317 (6)	−0.0114 (5)	0.0054 (6)	−0.0001 (4)
O4	0.0349 (5)	0.0378 (6)	0.0470 (7)	0.0080 (5)	0.0216 (5)	0.0181 (5)
N1	0.0396 (7)	0.0303 (7)	0.0425 (8)	0.0008 (5)	0.0223 (7)	−0.0016 (5)
N2	0.0311 (6)	0.0278 (6)	0.0303 (6)	−0.0003 (5)	0.0121 (5)	−0.0022 (5)
C1	0.0600 (12)	0.0448 (11)	0.0648 (12)	0.0045 (9)	0.0380 (10)	−0.0088 (9)
C2	0.0752 (15)	0.0628 (14)	0.0839 (16)	0.0239 (12)	0.0573 (14)	0.0053 (12)
C3	0.0444 (11)	0.0698 (14)	0.0799 (16)	0.0211 (10)	0.0403 (12)	0.0285 (12)
C4	0.0306 (8)	0.0519 (11)	0.0512 (10)	0.0054 (7)	0.0182 (8)	0.0233 (8)
C5	0.0272 (8)	0.0749 (15)	0.0629 (12)	−0.0076 (9)	0.0076 (9)	0.0273 (11)
C6	0.0416 (9)	0.0677 (14)	0.0484 (10)	−0.0247 (10)	−0.0015 (9)	0.0075 (10)
C7	0.0443 (9)	0.0438 (10)	0.0339 (8)	−0.0147 (8)	0.0041 (7)	0.0006 (7)
C8	0.0755 (14)	0.0480 (12)	0.0442 (10)	−0.0217 (10)	0.0114 (10)	−0.0191 (9)
C9	0.0798 (15)	0.0431 (11)	0.0507 (11)	−0.0004 (10)	0.0276 (11)	−0.0178 (9)
C10	0.0481 (9)	0.0356 (9)	0.0394 (8)	0.0042 (7)	0.0186 (7)	−0.0049 (7)
C11	0.0291 (7)	0.0323 (8)	0.0364 (8)	0.0009 (6)	0.0137 (6)	0.0103 (6)
C12	0.0313 (7)	0.0298 (7)	0.0283 (7)	−0.0041 (6)	0.0078 (6)	0.0036 (6)

### Geometric parameters (Å, °)

**Table:**

Mg1—O4	2.0333 (13)	C1—C2	1.403 (3)
Mg1—O2W	2.0423 (12)	C1—H1	0.9300
Mg1—O3W	2.0439 (13)	C2—C3	1.350 (3)
Mg1—O1W	2.0864 (12)	C2—H2	0.9300
Mg1—N2	2.2096 (14)	C3—C4	1.401 (3)
Mg1—N1	2.2335 (15)	C3—H3	0.9300
S1—O1	1.4545 (12)	C4—C11	1.411 (2)
S1—O4	1.4659 (11)	C4—C5	1.427 (3)
S1—O3	1.4702 (11)	C5—C6	1.339 (3)
S1—O2	1.4929 (12)	C5—H5	0.9300
O1W—H1W1	0.864 (9)	C6—C7	1.435 (3)
O1W—H1W2	0.859 (19)	C6—H6	0.9300
O2W—H2W1	0.850 (9)	C7—C8	1.397 (3)
O2W—H2W2	0.852 (9)	C7—C12	1.407 (2)
O3W—H3W1	0.848 (9)	C8—C9	1.361 (3)
O3W—H3W2	0.843 (9)	C8—H8	0.9300
N1—C1	1.327 (2)	C9—C10	1.397 (3)
N1—C11	1.354 (2)	C9—H9	0.9300
N2—C10	1.321 (2)	C10—H10	0.9300
N2—C12	1.3607 (19)	C11—C12	1.437 (2)
			
O4—Mg1—O2W	95.32 (5)	N1—C1—C2	122.7 (2)
O4—Mg1—O3W	102.08 (6)	N1—C1—H1	118.7
O2W—Mg1—O3W	85.12 (5)	C2—C1—H1	118.7
O4—Mg1—O1W	88.53 (5)	C3—C2—C1	119.86 (19)
O2W—Mg1—O1W	174.49 (5)	C3—C2—H2	120.1
O3W—Mg1—O1W	90.23 (5)	C1—C2—H2	120.1
O4—Mg1—N2	90.46 (5)	C2—C3—C4	119.51 (17)
O2W—Mg1—N2	86.69 (5)	C2—C3—H3	120.2
O3W—Mg1—N2	165.60 (6)	C4—C3—H3	120.2
O1W—Mg1—N2	97.24 (5)	C3—C4—C11	117.25 (18)
O4—Mg1—N1	162.63 (6)	C3—C4—C5	123.33 (18)
O2W—Mg1—N1	92.99 (6)	C11—C4—C5	119.42 (19)
O3W—Mg1—N1	93.80 (6)	C6—C5—C4	121.16 (17)
O1W—Mg1—N1	84.35 (5)	C6—C5—H5	119.4
N2—Mg1—N1	74.81 (5)	C4—C5—H5	119.4
O1—S1—O4	110.49 (8)	C5—C6—C7	121.38 (18)
O1—S1—O3	110.20 (7)	C5—C6—H6	119.3
O4—S1—O3	109.56 (7)	C7—C6—H6	119.3
O1—S1—O2	109.44 (8)	C8—C7—C12	116.99 (17)
O4—S1—O2	108.51 (6)	C8—C7—C6	124.00 (18)
O3—S1—O2	108.60 (7)	C12—C7—C6	119.01 (18)
Mg1—O1W—H1W1	119.2 (13)	C9—C8—C7	120.31 (17)
Mg1—O1W—H1W2	105.4 (13)	C9—C8—H8	119.8
H1W1—O1W—H1W2	106.0 (12)	C7—C8—H8	119.8
Mg1—O2W—H2W1	126.3 (12)	C8—C9—C10	118.85 (18)
Mg1—O2W—H2W2	123.7 (12)	C8—C9—H9	120.6
H2W1—O2W—H2W2	108.3 (13)	C10—C9—H9	120.6
Mg1—O3W—H3W1	119.9 (12)	N2—C10—C9	123.16 (17)
Mg1—O3W—H3W2	124.3 (13)	N2—C10—H10	118.4
H3W1—O3W—H3W2	110.3 (13)	C9—C10—H10	118.4
S1—O4—Mg1	141.98 (7)	N1—C11—C4	123.02 (16)
C1—N1—C11	117.67 (15)	N1—C11—C12	117.63 (13)
C1—N1—Mg1	127.63 (13)	C4—C11—C12	119.34 (16)
C11—N1—Mg1	114.61 (10)	N2—C12—C7	122.64 (15)
C10—N2—C12	117.98 (14)	N2—C12—C11	117.74 (13)
C10—N2—Mg1	126.81 (11)	C7—C12—C11	119.62 (15)
C12—N2—Mg1	115.12 (10)		
			
O1—S1—O4—Mg1	−99.19 (14)	C2—C3—C4—C5	179.9 (2)
O3—S1—O4—Mg1	139.21 (13)	C3—C4—C5—C6	179.20 (19)
O2—S1—O4—Mg1	20.79 (15)	C11—C4—C5—C6	−1.3 (3)
O2W—Mg1—O4—S1	166.33 (13)	C4—C5—C6—C7	0.1 (3)
O3W—Mg1—O4—S1	80.20 (14)	C5—C6—C7—C8	−177.5 (2)
O1W—Mg1—O4—S1	−9.73 (14)	C5—C6—C7—C12	2.2 (3)
N2—Mg1—O4—S1	−106.96 (14)	C12—C7—C8—C9	1.0 (3)
N1—Mg1—O4—S1	−75.4 (2)	C6—C7—C8—C9	−179.3 (2)
O4—Mg1—N1—C1	146.11 (19)	C7—C8—C9—C10	1.1 (3)
O2W—Mg1—N1—C1	−95.33 (16)	C12—N2—C10—C9	−0.5 (2)
O3W—Mg1—N1—C1	−10.02 (16)	Mg1—N2—C10—C9	−176.88 (14)
O1W—Mg1—N1—C1	79.84 (16)	C8—C9—C10—N2	−1.4 (3)
N2—Mg1—N1—C1	178.93 (17)	C1—N1—C11—C4	0.5 (2)
O4—Mg1—N1—C11	−30.2 (2)	Mg1—N1—C11—C4	177.26 (12)
O2W—Mg1—N1—C11	88.34 (11)	C1—N1—C11—C12	179.79 (16)
O3W—Mg1—N1—C11	173.64 (11)	Mg1—N1—C11—C12	−3.49 (17)
O1W—Mg1—N1—C11	−96.50 (11)	C3—C4—C11—N1	−1.1 (2)
N2—Mg1—N1—C11	2.60 (10)	C5—C4—C11—N1	179.40 (15)
O4—Mg1—N2—C10	−14.19 (14)	C3—C4—C11—C12	179.68 (15)
O2W—Mg1—N2—C10	81.12 (14)	C5—C4—C11—C12	0.2 (2)
O3W—Mg1—N2—C10	136.5 (2)	C10—N2—C12—C7	2.7 (2)
O1W—Mg1—N2—C10	−102.77 (13)	Mg1—N2—C12—C7	179.56 (12)
N1—Mg1—N2—C10	175.12 (14)	C10—N2—C12—C11	−176.75 (14)
O4—Mg1—N2—C12	169.30 (10)	Mg1—N2—C12—C11	0.08 (17)
O2W—Mg1—N2—C12	−95.39 (11)	C8—C7—C12—N2	−3.0 (2)
O3W—Mg1—N2—C12	−40.0 (3)	C6—C7—C12—N2	177.28 (15)
O1W—Mg1—N2—C12	80.72 (11)	C8—C7—C12—C11	176.47 (16)
N1—Mg1—N2—C12	−1.39 (10)	C6—C7—C12—C11	−3.3 (2)
C11—N1—C1—C2	0.7 (3)	N1—C11—C12—N2	2.3 (2)
Mg1—N1—C1—C2	−175.54 (16)	C4—C11—C12—N2	−178.38 (14)
N1—C1—C2—C3	−1.4 (3)	N1—C11—C12—C7	−177.16 (14)
C1—C2—C3—C4	0.8 (3)	C4—C11—C12—C7	2.1 (2)
C2—C3—C4—C11	0.4 (3)		

### Hydrogen-bond geometry (Å, °)

**Table:**

*D*—H···*A*	*D*—H	H···*A*	*D*···*A*	*D*—H···*A*
O2W—H2W1···O3^i^	0.850 (9)	1.891 (11)	2.7170 (17)	163.8 (16)
O3W—H3W1···O2^ii^	0.848 (9)	1.906 (10)	2.7375 (17)	166.2 (17)
O1W—H1W1···O1^ii^	0.864 (9)	1.862 (10)	2.7235 (17)	174.8 (17)
O2W—H2W2···O2^iii^	0.852 (9)	1.873 (10)	2.7232 (17)	175.4 (19)
O1W—H1W2···O2	0.859 (19)	1.862 (19)	2.7030 (17)	166.0 (19)
O3W—H3W2···O3^iii^	0.843 (9)	1.965 (10)	2.7961 (19)	168.5 (17)

Symmetry codes: (i) −*x*, −*y*+1, −*z*+1; (ii) −*x*, −*y*, −*z*+1; (iii) *x*, −*y*+1/2, *z*−1/2.
